# Impact of PI-RADS v2 on indication of prostate biopsy

**DOI:** 10.1590/S1677-5538.IBJU.2018.0564

**Published:** 2019-07-27

**Authors:** George de Queiroz Rozas, Lucas Scatigno Saad, Homero José de Farias e Melo, Henrique Armando Azevedo Gabrielle, Jacob Szejnfeld

**Affiliations:** 1 Departamento de Diagnóstico por Imagem, Universidade Federal de São Paulo - USP, São Paulo, SP, Brasil;; 2 Faculdade de Ciências Médicas da Santa Casa de São Paulo, São Paulo, SP, Brasil;; 3 CURA Imagem e Diagnóstico, São Paulo, SP, Brasil

**Keywords:** Prostate, Biopsy, Magnetic Resonance Imaging

## Abstract

**Objectives:**

To identify the group of patients who could safely avoid prostate biopsy based on the findings of multiparametric prostate resonance imaging (MRmp), parameterized with PI-RADS v2, using prostate biopsy as reference test and to assess the sensitivity and specificity of mpMR in identifying clinically significant prostate cancer using prostate biopsy as a reference test.

**Patients and Methods:**

Three hundred and forty two patients with suspected prostate cancer were evaluated with mpMR and prostate biopsy. Agreement between imaging findings and histopathological findings was assessed using the Kappa index. The accuracy of mpMR in relation to biopsy was assessed by calculations of sensitivity, specificity, positive predictive value (PPV) and negative predictive value (NPV).

**Results:**

A total of 342 biopsies were performed. In 201 (61.4%), mpMR had a negative result for cancer, which was confirmed on biopsy in 182 (53%) of the cases, 17 (4.9%) presented non-clinically significant cancer and only 2 (0.5%) clinically significant cancer. 131 (38.3%) patients had a positive biopsy. Clinically significant cancer corresponded to 83 (34.2%), of which 81 (97.5%) had a positive result in mpMR. Considering only the clinically significant cancers the mpMR had a sensitivity of 97.6%, specificity of 76.8%, PPV 57.4% and VPN of 99%.

**Conclusions:**

mpMR is a useful tool to safely identify which patients at risk for prostate cancer need to undergo biopsy and has high sensitivity and specificity in identifying clinically significant prostate cancer.

## INTRODUCTION

Prostate cancer (PCa) is the second more common type of cancer among men (1). According to the World Health Organization (WHO), early detection of a cancer involves two strategic approaches: screening and early diagnosis.

Main obstacles to early diagnosis of PCa include the inability of rectal exam and PSA to distinguish benign, and subclinical conditions, and clinical significant prostate cancer, with underdiagnosis in up to 40 to 60% of patients (2, 3).

PCa algorithm differs from others of other solig organ cancers, in which an image exam identifies patients who need evaluation by biopsy. During PCa propedeutics, biopsy is indicated to all patients with elevated PSA, suspected rectal exam or significant familial history (4–7).

The use of a diagnostic protocol, in which an image exam defines which patients with elevated PSA and/or altered rectal exam should be submitted to further investigation could significantly reduce the number of biopsies and improve diagnostic accuracy of current propedeutics (8, 9).

Magnetic ressonance image (MRI) is the exam of image of choice for early diagnosis, location and staging of prostate cancer (10–14). Multiparametric resonance of prostate (mpMR) uses functional and anatomic sequences, such as high resolution balanced T2 sequences in three orthogonal plans, and sequences of diffusion and perfusion, that evaluates not only tecidual anatomy but also volumetry, cellularity and tissue vasculature, resulting in a better significant accuracy of the method (15).

MpMRp detects high risk disease and systematically disclose low risk tumors, making it a very interesting potential screening test (16, 17).

European Society of Urogenital Radiology (ESUR) published in 2012 a series of guidelines recommending the interpretation of MpMRp images and a structured graph to describe and obtain a report called PI-RADS - Prostate Imaging Reporting and Data System. In 2015, the American College of Radiologists, EUSR and AdMe Tech Foundation improved and updated PI-RADS to version 2 (v2) (18).

PI-RADS v2 uses a scale based on probability that the combination of findings in T2 sequences, diffusion and contrast is related to the presence of clinically significant prostate cancer (19).

According to PI-RADS v2, a high score indicates the presence of a tumor with higher dimensions, homogeneous low signal, significant restriction to dfusion and early highlight after dye administration (20, 21).

In this context, we propose this paper with the main goal to identify which group of patients could, in a safe environment, avoid the use of prostate biopsy based on the results of MpMRp according to PI-RADS v2, using prostate biopsy as reference test. The secondary objective was to evaluate the sensitivity and specificity of Mpmrp in the identification of clinically significant prostate cancer, using prostate biopsy as reference test.

## MATERIAL AND METHODS

This is a retrospective cohort sectional study performed from June 2015 to August 2016, with patients from the Cura Clinic, São Paulo, Brazil.

The study was approved by the Ethics and Research Committee of UNIFESP#0369/2017.

### Patients

From June 2015 to August 2016 we identified in our data bank the results of 764 MpMRp of prostate in patients with 43 to 85 years old.

The exams were ordered due to clinical suspicion of prostate cancer. The patients presented at least one of the following criteria: suspected rectal exam, elevated PSA (> 4.0nd/dL), familiar history of PCa and prostate ultrasound showing focal lesion. All patients were referred to prostate biopsy by their urologist.

**Figure 1:**
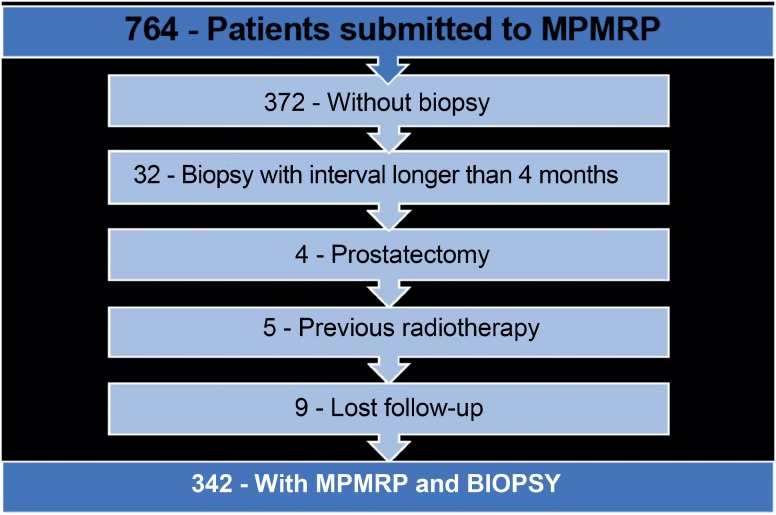
Algorithm of patients under study.

We revised the chart of 764 patients submitted to MpMRp and identified 342 that were submitted to prostate biopsy in up to 4 months after the image exam. Exclusion criteria included: prostate biopsy after more than 4 months, previous prostatectomy and previous radiotherapy (Figure-1).

## MpMRp

### Equipment

Patients were submitted to MpMRp exam in 3 Tesla (T) equipments, Verio and Skyra, Siemens, Germany, with coil of 8 channels.

### Protocol of image acquisition

All patients were submitted to the same MpMRp protocol.

The multiparametric exams included balanced images in T1, T2, diffusion (DW), and dynamic contrast with listed parameters in Table-1.

**Table 1:** MpMRp parameters.

Image protocol	Sequence	RT (ms)	ET (ms)	FA (0)	b value	Cut width (mm)	FOV (mm)	Matrix	Temporal resolution
T1 AXIAL	SE	550	9.5	131	--	3	150	256	--
T2 SAGITAL	TSE	3790	114	160	--	3	160	256	--
T2 CORONAL	TSE	3560	114	160	--	3	160	256	--
T2 AXIAL	TSE	3930	124	160	--	3	150	256	--
T2 AXIAL FAT SAT	TSE	3930	124	160	--	3	150	256	--
DW AXIAL	SSEP with fat suppresion	5200	68		0, 100, 400, 800		320	160	
Dye	3D GRE	3.8	1.5	10	--	1.6	200	288	2.5

**RT** = repetition time; **ET** = echo time; **FA** = flip angle; **FOV** = field of view; **DW** = diffusion weighted; **SE** = spin echo; **TSE** = turbo spin echo; **SSEP** = 3D GRE; **3D** = gradient echo

### Image Analysis

Mpmrp exams were analysed with a specific Syngovia software at work stations.

Images of all patients were evaluated by two radiologists (consensus) with 15 years (J.S) and 4 years (G.Q.R.) of experience in prostate images, without knowing the results of prostate biopsy. Both radiologists were aware of the indications of the MpMRp exam.

PI-RADS v2 was used to classify the prostates according to the probability of clinically significant prostate cancer: 1) Highly unlikely; 2) Unlikely; 3) Undetermined; 4) Probable; 5) Highly probable.

### Histologic Evaluation

Prostate biopsies were performed with cognitive image fusion, and in media 4 fragments were obtained in suspected areas identified by Mpmrp and random fragments from other areas of prostate. In patients without identification by MpMRp of suspected areas, biopsy was indicated by clinical and biochemical criteria, and 18 fragments were obtained randomly. The samples were sent to pathology exam.

Positive samples of prostate adenocarcinoma were classified according to Gleason Score modified by ISUP in 2005* (22).

Clinically significant disease was defined as the presence of Gleason 4 as primary of secondary pattern, given the associated risk of extra-prostatic disease and biochemical recidive associated to this pattern (23).

PI-RADS was dicotomized in order to correlate biopsy and Mpmrp results. PI-RADS 1 and 2 results were considered negative for cancer, and Pi-RADS 3, 4, and 5 results were considere positive. Next, Mpmrp results were grouped according to biopsy results: positive, negative biopsy and with both groups.

## Statistical analysis

It was performed descriptive analysis of demographic characteristics of patients and calculated the prevalence of PCa with a Confidence Interval (CI) of 95%. To evaluate the accuracy of MpMRp exam in relation to prostate biopsy, it was used the calculation of sensitivity, specificity, positive predictive value (PPV), and negative predictive value (NPV) with their respective CI 95%.

In order to evaluate the concordance grade between the image results and the histological analysis, it was used the kappa index (k) k = Po-Pe/1-Pe. Po is the proportion of observed agreement and Pe is the proportion of expected agreements. The following values of k were considered: k < 0.00: bad; 0.00 to 0.20: weak; 0.21 to 0.40: regular; 0.41 to 0.60: moderate; 0.61 to 0.80: substantial; 0.81 to 0.99: almost perfect.

## RESULTS

**Figure 2:**
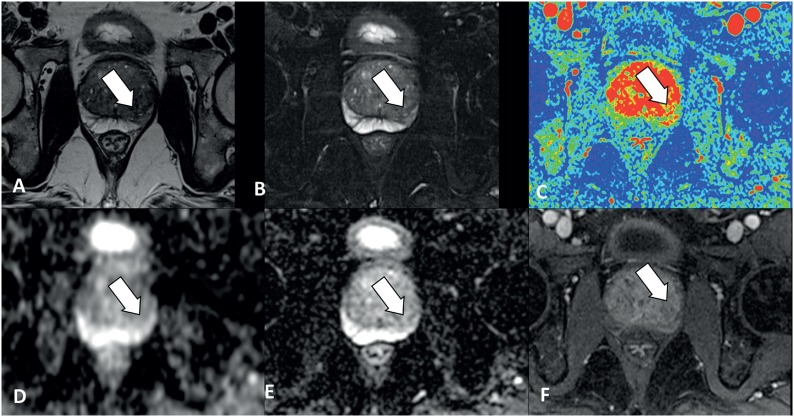
Forty five-year-old patient with prostatitis. PI-RADS 2. **A)** T2 and **B)** T2 Fat Sat show areas of low homogeneous linear and wedge-shaped signals. **C)** T1 dynamic after contrast with color map showing discreet enhancement. **D)** ADC map and **E)** DWI without diffusion restriction. **F)** T1 dynamic after contrast shows discrete highlight in the corresponding area.

Three hundred and forty two prostate biopsies were performed. In 201 (61.4%), MpMRp was negative for cancer, and in 182 (53.2%) the biopsy was also negative, 17 (4.9%) showed non clinically significant tumor and in only 2 biopsies (0.5%) there was clinically significant prostate cancer (Table-2 and Figure-2).

**Table 2:** Relation between the result of mpMR and biopsy result.

342 biopsies					
mpMRp (-): 201 (100%)			mpMRp (+): 141 (100%)		
NC: 182 (90.5%)	NCSC: 17 (8.4%)	CSC: 2 (0.9%)	NC: 29 (20.5%)	NCSC: 31 (21.9%)	CSC: 81 (57.4%)

**Bx** = biopsy; M**pMRp** = multiparametric magnetic ressonance of prostate; **NC** = no cancer; **NCSC** = non clinically significant cancer; **CSC** = clinically significant cancer

One hundred thirty one patients (38.3%) had positive bipsies and 211 (61.6%) had at least 1 negative biopsy.

**Figure 3:**
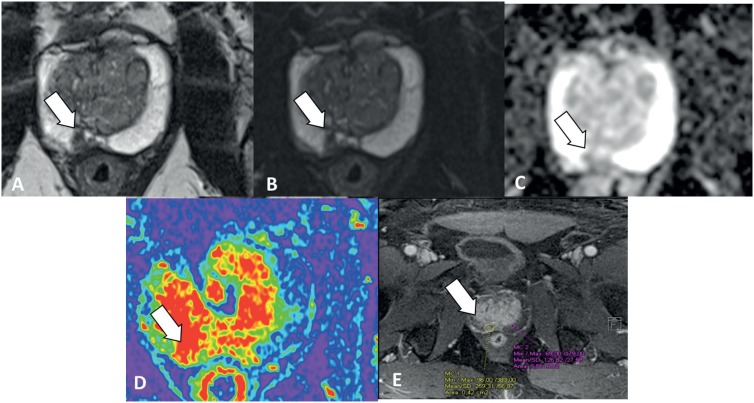
Fifty-year-old patient with clinically significant cancer. **PI-RADS 4 - A)** T2 and **B)** T2 Fat Sat show a homogeneous low signal nodule measuring 1 cm. **C)** ADC map shows focal area of restriction to in the same place. **D)** T1 dynamic after contrast with color map and E, dynamic T1 after contrast showing focal area of enhancement.

Prevalence of clinically significant cancer, according to previously described, was 83 (34.2%) of 342 patients (Figure-3).

**Figure 4:**
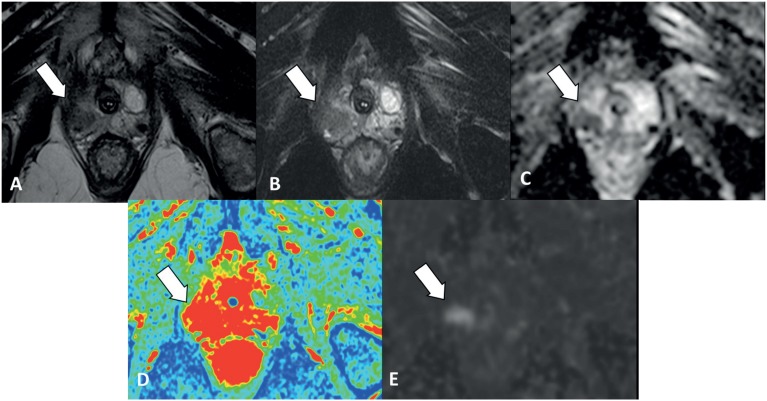
56 year old patient with clinically significant cancer. PI-RADS 5. **A)** T2 and **B)** T2 Fat Sat showing a homogeneous low signal nodule, measuring 1.8 cm. **C)** ADC map shows focal area of restriction diffusion in the same place. **D)** T1 dynamic after contrast with color map noting capture area local and **E)** diffusion sequence showing hypersignal / diffusion restriction.

Among the 83 patients with clinically significant tumor, 81 (97.5%) had positive MpMRp results and only 2 (2.5%) had a negative result. The two ptients were classified as Gleason 7 at biopsy (Figure-4).

Considering all tumors, including those non clinically significant (Gleason 6), MpMRp was negative in 19 (14.5%) from 131 positive patients, being 17 Gleason 6 and only 2 Gleason 7.

MpMRp was PI-RADS 3 (undetermined) in 14 (10.6%) of 131 positive biopsies.

**Figure 5:**
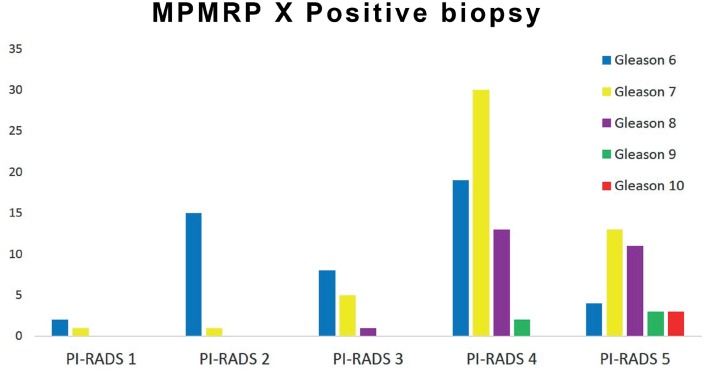
Distribution of the PI-RADS classification according to Gleason in a patient with positive biopsy.

MpMRp PI-RADS 4 and 5 (probable and highly probable presence of tumor) was observed in 98 (74.5%) of 131 positive biopsies (Figure-5).

Of the 211 patients with negative biopsy, 182 (88%) had MpMRp negative results (PI-RADS 1 - 15 patients, and PI-RADS 2 - 167 patients); among these 211 patients, 29 (12%) had MpMRp positive results.

No MpMRp was considered PI-RADS 5 (highly probable tumor) in the group of patients with negative biopsy.

PI-RADS 3 total MpMRp results (undetermined), including both groups with positive and negative biopsies, included 25 (7.3%) of 342 patients, of whom 14 had positive biopsies and 11 negative.

MpMRp PI-RADS 4 total results (probable cancer), including positive and negative biopsy groups, was 82 (23.9%) of 342, among whom 64 had positive biopsies and 18 negative (Table-3).

Concordance kappa coeficiente between both methods varied from moderate to substantial, allowing the comparison of both methods.

Considering all cancers (non clinically significant and clinically significant), sensitivity of MpMRp was 85.5% and specificity 86.3%, with PPV of 80% and NPV of 90.5%.

Considering only clinically significant tumors, sensitivity of MpMRp was 97.6% and specificity 76.8%, and PPV of 57.4% and NPV of 99% (Table-4).

**Table 4:** Sensitivity, specificity, PVV and NPV of MpMRp to detect prostate cancer, considering all tumors and only those clinically significant.

Biopsy	MpMRp		Total	Kappa CI (95%)	Sens CI (95%)	Spec CI (95%)	PPV CI (95%)	NPV CI (95%)
	No	Yes						
CA total, n (%)				0,707	85,5	86,3	79.4	90.5
No	182 (53.2)	29 (8.5)	211 (61.7)	(0,631; 0.783)	(78.3; 91)	(80.9; 90.6)	(71.8; 85.8)	(85.6; 94.2)
Yes	19 (5.6)	112 (32.7)	131 (38.3)					
CSC, n (%)				0,601	97.6	76.8	57.4	99
No	199 (58.2)	60 (17.5)	259 (75.7)	(0,519; 0.683)	(91.6; 99.7)	(71.2; 81.8)	(48.8; 65.7)	(96.5; 99.9)
Yes	2 (0.6)	81 (23.7)	83 (24.3)					
**Total**	**201 (58.8)**	**141 (41.2)**	**342 (100)**					

## DISCUSSION

The introduction of the MpMRp exam in the algorithm of PCa diagnosis as screening test, to define which patients with suspected tumor must be submitted to biopsy, can significantly change the current scenario (8, 9).

According to our results of all patients with negative MpMRp (PI-RADS 1 and 2), only 2 had clinically significant tumors at biopsy. A total of 199 were truly negative clinically significant cancers and only 2 were false negatives.

This finding proves the excellent NPV of MpMRp; in the studied population it could have prevented safely the use of biopsy in up to 181 patients of a total of 211, corresponding to 58% of patients (14, 24).

This value is much more elevated than the one recently described in a significant study and is probably related to the value of PSA used to indicate the biopsy. In that study, it was used a value of ≥ 15ng/mL (9).

Also, 52 of the 211 patients with negative biopsy have had a biopsy in another occasion due to clinical indication; 16 had been submitted to 2 biopsies in other occasions, 7 in three other moments and 2 patients were submitted to 4 biopsies in other moments, according to charts review.

In the opposite side of PI-RADS scale (PI-RADS 5, or highly suspected PCa), a total of 34 exams with this result all had positive biopsies, and only 4 patients had a Gleason 6 score; all others (30) (88%) had Gleason 7 to 10, corresponding to clinically significant tumors.

As mentioned before, according to PI-RADS, a high score indicates the presence of tumors with higher dimensions, low homogeneous sign, significant restriction to diffusion and early highlight after contrast injection (20, 21, 25–27).

These literature data are in accordance to our results of PI-RADS 5 and 4 MpMRp.

64 patients had PI-RADS 4 MpMRp results; 45 of those (70.1%) had clinically significant tumors, with Gleason 7 to 10, and 19 (21.9%) had Gleason 6, confirming the higher tendency of PI-RADS 4 correlates to the incidence of clinically significant tumors.

As PI-RADS Mpmrp results diminished (characterizing less evident tumor at image exam), the probability of tumor also diminished, as well as the incidence of clinically significant cancers.

Twenty five exams were Mpmrp PI-RADS 3. Of these, 14 had positive biopsies and 11 negative. Of the 14 positive confirmed biopsies, 8 were classified as Gleason 6, 5 as Gleason 7 and 1 as Gleason 8. These results show that there is a balanced distribution of tumors and no tumor patients, in accordance to the undetermined cathegorization of PI-RADS 3 results.

However, a few patients with PI-RADS 3 and 4 at MpMRp showed benign biopsy, revealing a lower PPV of MpMRp, in accordance to literature (14, 15).

Another significant result of our study in that all patients with clinically significant tumors had PI-RADS 3 to 5 results, except 2/59 (3%), delimiting a cut-off value for the indication of biopsy.

In our sample, there were 15 positive biopsies with negative MpMRp; however, all tumors were non clinically significant Gleason 6 cancers.

Previous MpMRp to prostate biopsy in a patient with clinical suspicion of prostate cancer, without the identification of a suspected focus, with PI-RADS 1 or 2 results, would imply in a close follow-up of this patient, with regular clinical and biochemical reevaluation, and possibly a new MpMRp (28).

Our study has some limitations. First, this is a retrospective study. Another limitation is the use of prostate biopsy as reference and not other histological confirmation methods such as prostatectomy. According to some papers, histological analysis of biopsy of prostate tissue guided by US underestimate the results of Gleason score in 26 to 41% of patients, in comparison to prostatectomy (4–7).

However, we chose biopsy results for this histological analysis since it is more used in daily clinical practice, reproducing more reliably most daily situations of the urologist. Another advantage of biopsy in relation to prostatectomy as reference pattern is the possibility to include in the sampling and analysis of Mpmrp all types of tumors, including those with lower Gleason score, non clinically significant, without indication of prostatectomy.

Also, if we had used samples of prostatectomy as reference pattern, the number of non clinically significant tumors certainly would be low in the analysed sample, restricting the analysis of the presence of tumors at MpMRp. Therefore, we would not identify one important characteristic of this image exam, the tendency to neglect low Gleason, non clinically significant tumors (29, 30).

Another limitation of our work was the comparison between the diagnostic methods, performed at the patient level and not of the region of the prostate or of the specific lesion. However, the determination of PI-RADS to identify the index lesion and the use of cognitive fusion with the MRI images during biopsy tend to minimize possible discrepancies, since the index lesion of Mpmrp would correspond to the focus of higher Gleason score at biopsy.

## CONCLUSIONS

MpMRp is a useful tool to safely identify which patients can be excluded for biopsy and remain without active surveillance/follow-up, due to its high NPV.

MpMRp has a high sensitivity and specific to identify clinically significant prostate tumor.
